# *In vitro* and *in vivo* antimalarial activities of the ethanol extract of *Erythrina sigmoidea* stem bark used for the treatment of malaria in the Western Region of Cameroon

**DOI:** 10.3389/fpara.2024.1359442

**Published:** 2024-04-09

**Authors:** Tientcheu Noutong Jemimah Sandra, Noumedem Anangmo Christelle Nadia, Yamssi Cedric, Gamago Nkadeu Guy-Armand, Mounvera Abdel Azizi, Ngouyamsa Nsapkain Aboubakar Sidiki, Tako Djimefo Alex Kevin, Vincent Khan Payne, Haibo Hu

**Affiliations:** ^1^ Department of Animal Biology Faculty of Science, University of Dschang, Dschang, Cameroon; ^2^ Laboratory of Tropical and Emerging Infectious Diseases, Dschang, Cameroon; ^3^ Department of Microbiology, Hematology and Immunology Faculty of Medicine and Pharmaceutical Sciences, University of Dschang, Dschang, Cameroon; ^4^ Department of Biomedical Sciences, Faculty of Health Sciences, University of Bamenda, Bambili, Cameroon; ^5^ Department of Animal Organisms, Faculty of Science, University of Douala, Douala, Cameroon; ^6^ National Engineering Research Center for Modernization of Traditional Chinese Medicine – Hakka Medical Resources Branch, Gannan Medical University, Ganzhou, China

**Keywords:** antiplasmodial, plasmodium, *Erythrina sigmoidea*, *in vivo*, *in vitro*, Cameroon

## Abstract

**Background:**

Malaria is one of the leading causes of morbidity and/or mortality in tropical Africa. The spread and development of resistance to chemical antimalarial drugs and the relatively high cost of the latter are problems associated with malaria control and are reasons to promote the use of plants to meet healthcare needs to treat malaria. The aim of this study was to evaluate antiplasmodial activities of extracts of *Erythrina sigmoidea* (Mah quat), which is traditionally used for the treatment of malaria in the western region of Cameroon.

**Material and methods:**

The ethanol extract of *E. sigmoidea* stem bark was obtained through the maceration process using 95% ethanol, while the aqueous extract was prepared by infusion. The *in vitro* antiplasmodial effect of extracts against *P. falciparum* chloroquine-sensitive (3D7) and chloroquine-resistant (Dd2) strains was determined using the Trager and Jensen method. On the other hand, the *in vivo* antimalarial activity of the extract was evaluated in mice infected with *Plasmodium berghei* strain NK65 using the Peters’ 4-day suppressive test and Ryley test (curative test). A total of 36 mice were used, subdivided into six groups of six mice each: one normal control, a negative control, a positive control, and three other groups for the tested product. Blood samples were collected on the 10th day of each test for hematological parameters.

**Results:**

The aqueous extract had an *in vitro* antiplasmodial activity against the chloroquine-sensitive strain with an IC_50_ of 29.51 ± 3.63 µg/mL and against the chloroquine-resistant strain with an IC_50_ of 35.23 ± 3.17 µg/mL. The highest *in vitro* antiplasmodial activity was observed with the ethanol extract against the chloroquine-sensitive strain with an IC_50_ of 6.44 ± 0.08 µg/mL and against the chloroquine-resistant strain with an IC_50_ of 7.53 ± 0.22 µg/mL. The ethanol extract demonstrated suppressive activity *in vivo* with reduction rates of 87.69%, 86.79%, and 81.08% at doses of 500 mg/kg, 250 mg/kg, and 125 mg/kg, respectively; and curative activity *in vivo* with reduction rates of 80%, 78.5%, and 77.5% at doses of 500 mg/kg, 250 mg/kg, and 125 mg/kg, respectively. The number of white blood cells in the negative control (44.55 ± 5.02 10^3^/µL) was higher compared to the other groups. As for the red blood cells, we observed a massive destruction of the latter in the infected and untreated group (5.82 ± 1.50 10^6^/µL) compared to the infected and ethanol extract-treated groups (8.74 ± 1.57 10^6^/µL for 500 mg/kg, 7.54 ± 1.77 10^6^/µL for 250 mg/kg, and 8.9 ± 1.50 10^6^/µL for 125 mg/kg).

**Conclusion:**

This study provides scientific data on the use of *E. sigmoidea* by the local population for the treatment of malaria. It shows that *E. sigmoidea* has antiplasmodial activity, and we also see that there are differences between the parameters that we have in the treated groups and those of the untreated group. However, toxicity tests are necessary to assess its safety.

## 1 Introduction

Especially in sub-Saharan Africa, Asia, and Latin America, malaria is a serious tropical illness with a high morbidity and fatality rate (Kolawole et al., 2023). A parasite known as Plasmodium is the cause of malaria and is spread by mosquito bites. There are five species of Plasmodium that people can contract and spread (Sato, 2021). *Plasmodium falciparum* is mostly responsible for severe illness. The human body’s liver is where parasites first proliferate, followed by erythrocytes. Fever, headaches, and vomiting are among the symptoms of malaria that often manifest 10 to 15 days following the mosquito bite (Balaji et al., 2020). Malaria can soon become fatal if left untreated because it can cut off blood flow to essential organs.

In 2019, the World Health Organization (WHO) estimated that, worldwide, approximately 241 million cases and 627,000 deaths were due to malaria (Liu et al., 2022). These increases are mainly observed in sub-Saharan Africa where there are 12% more deaths than in 2020 (Murray et al., 2007). In sub-Saharan Africa particularly, malaria remains the primary medical concern. The vulnerable groups are mainly children under 5 years old and pregnant women (Gilmartin et al., 2021). The RTS, S/AS01 malaria vaccine implemented in Ghana, Kenya, and Malawi by the WHO in children from the age of 5 months has the specific aim of reducing the parasitic load and not of actually preventing the disease. In Cameroon, malaria constitutes a serious public health problem despite an improvement in certain indicators and the surveillance system (Haidara et al., 2016).

In Cameroon, malaria is also a major health burden for families (Antonio-Nkondjio et al., 2019). Malaria has been managed by an integrated approach that includes medication treatment and vector control (Shiff, 2002). Presently, the main medications used to treat malaria are artemisinin-based combination treatments (ACTs) (Ouji et al., 2018). On the other hand, ACTs are expensive. Regrettably, there have been indications of rising drug resistance to derivatives of artemisinin (Wongsrichanalai and Sibley, 2013). The steady increase in resistance to the majority of available antimalarial drugs and the ability of malaria vectors to resist insecticides are responsible for the re-emergence of malaria in many parts of the world (Na-Bangchang and Karbwang, 2009). The alarming increase in malaria drug resistance coupled with vector resistance has led to the failure of the prevention and eradication program put in place (Sidiki et al., 2023). It is now established that natural products constitute a major and abundant source of anti-infective agents (Mahmoudvand et al., 2023).

In the past, traditional herbs had wide acceptance in the treatment of malaria. As early as the 17th century, infusion of the barks of plant species like Cinchona (Rubiaceae) were effective for the treatment of human malaria (Ceravolo et al., 2021). Over time, quinine has been isolated and characterized, making it an essential drug against malaria (Jones et al., 2015). Therefore, it is crucial to keep up the fight against malaria in Cameroon, and a number of scientists there are using ethnobotanical data to identify effective medicinal plants that could lead to the development of novel antimalarial drugs. In fact, researching medicinal plants is still an effective way to find novel medications. Medicinal herbs are the most cost-effective means of treatment in endemic areas, and substances with antiplasmodial activity include flavonoids, terpenoids, and alkaloids (Narayanan et al., 2019).

*Erythrina sigmoidea*, family Fabaceae (common name: Mah quat), stem bark is used by the local population of Foumban for the treatment of malaria, typhoid fever, helminthiasis, hepatitis, venereal diseases, conjunctivitis, piles, diarrhea, dysentery, malaria, microbia, bacteria, jaundice, and syphilis, and has many properties such as hallucinatory, aphrodisiac, diuretic, hemostatic, purgative, and tonic. This plant has shown anti-inflammatory, antibacterial, and anticholinergic activities based on phytochemical analysis (Narayanan et al., 2019). Another leguminous plant, *Bauhinia variegata*, showed marked antileishmanial activity with cell cycle arrest at the sub-G0/G1 phase. *B. variegata* was found to be more effective at a higher dose in declining parasite concentration in the spleen as compared to the lower dose (Kaur et al., 2021). The aim of this study was to evaluate the antiplasmodial activity of *E.* sigmoidea, a medicinal plant used for the treatment of malaria in the western region of Cameroon and to provide scientific evidence on its use as an antimalaria agent.

## 2 Material and methods

### 2.1 Collection and identification

The stem bark plant was harvested in Foumban West Region of Cameroon on November 2023 in the dry season. The following plant material were sent to the National Herbarium of Cameroon in Yaounde for identification: stem bark, leaves, seeds, and flowers. The plant was identified with Reference Number 8749/SRFCAM by Dr NGANTSOP.

### 2.2 Preparation of extracts

Ethanol extract was used for the extraction because during the ethnobotanical study, a traditional practitioner used palm wine (ethanol) for maceration or infusion to prepare this remedy. The aqueous extract was prepared by infusion while the ethanol extract was obtained using the procedure described by Wabo et al. (2011). Briefly, 100 g of powder was weighted using an electric balance (SF-400) and introduced into two 3-L containers. One liter of 95% ethanol and heated (100°C) distilled water were introduced in each container and the mixtures were stirred for 5 min. The mixture of ethanol was macerated for 72 h at room temperature, and that of the aqueous solution was left to cool down. The homogenates were first filtered using a 150-μm sieve and then with Whatman paper (No. 1). The filtrates obtained were introduced into an oven at 40°C for evaporation until aqueous and ethanol extracts were obtained.

### 2.3 *In vitro* antiplasmodial activity

In summary, fresh human red blood cells of group O+ were used to culture the chloroquine-sensitive Pf3D7 strains and the multi-resistant PfDd2 strains at 4% hematocrit in complete RPMI medium (Gibco, UK). This medium was supplemented with 25 mM HEPES (Gibco, UK), 0.50% Albumax I (Gibco, USA), 1× hypoxanthine (Gibco, USA), and 20 µg/mL gentamicin (Gibco, China). The cells were then incubated at 37°C in a humidified incubator with 92% N_2_, 5% CO_2_, and 3% O_2_. Using the Trager and Jensen method (Trager and Jensen, 1997), the chloroquine-sensitive (3D7) and chloroquine-resistant (Dd2) strains, frequently employed in drug sensitivity experiments, were used. The culture was carried out in Petri dishes and maintained in a humid atmosphere in an incubator. The culture medium in the Petri dishes in culture was renewed daily. The parasitemia of the cultured strain was checked on a May Grunwald-Giemsa-stained thin smear. The differential permeability of the old stages to D-Sorbitol was used to destroy them selectively by osmotic lysis, which makes it possible to obtain cultures enriched in ring stages. Briefly, 90 μL of the parasite solution that was synchronized at the ring stage with 2% parasitemia and 1% hematocrit was incubated with 10 μL of various concentrations of extracts, artemisinin, and chloroquine. The plates were incubated for 48 h. The antiplasmodial activity test of plant extracts, artemisinin, and chloroquine was carried out in 96-well flat-bottomed microplates based on the fluorescence of SYBR green I. The ability of SYBR green I to produce strong fluorescence only in the presence of DNA is the basis for its use in the assessment of cell proliferation. The absence of a cell nucleus inside human red blood cells in which the parasite proliferates allows specific monitoring of plasmodial growth using SYBR green I. One hour prior to the end of incubation, 100 μL of SYBR green was added. The fluorescence was measured using a fluorescence microplate reader at an excitation and emission wavelength of 485 and 538 nm, respectively. The resistance index (RI) was calculated using the formula (Kevin et al., 2023):


$$\text{RI}=\frac{\text{CI}50\;\text{extracted}\;\text{on}\;\text{PfDd}2}{\text{CI}50\;\text{extracted}\;\text{on}\;\text{Pf}3\text{D}7}$$

### 2.4 *In vivo* antiplasmodial activity

#### 2.4.1 Choice of the gavage solution doses

Approximately 650 g of *E. sigmoidea* plant powder (stem bark), as indicated by the traditional practitioner, was infused in 6 L of water for 15 min. The resulting mixture was cooled and filtered through Whatman No. 1 paper. Three glasses (the practitioner’s daily volume, which is approximately 1 L) were taken from the filtrate and evaporated in a ventilated oven at 40°C, giving a weight of approximately 16.5 g. Assuming that the average weight of a man is 70 kg, the dose of 235.71 mg/kg (16.5/70) = 250 mg/kg was obtained. This dose was divided by 2 and multiplied by 2 to get 125 mg/kg and 500 mg/kg, respectively.

#### 2.4.2 The Peters’ 4-day suppressive test

Mice (*Mus musculus*) from the Animal Facility of the Center for Schistosomiasis and Parasitology (CSP) in Yaoundé (at the sports stadium) was used in the *in vivo* tests. *Plasmodium berghei* NK 65 strain given by the American Type Culture Collection (ATCC) was used. Two weeks were taken for the acclimatization of the mice. The antimalarial activity of the extract was determined using Peters’ 4-day suppressive test (Peters, 1975). A total of 36 mice with an average weight of 25 ± 0.5 g were divided into six groups of six mice each. Mice were randomly inoculated with 1×10^7^ parasitized erythrocytes with *P. berghei* (except normal control group), stained with picric acid and dispatched into different groups. Three hours after infection, the first three groups received the ethanol extract of *E. sigmoidea* at doses of 125, 250, and 500 mg/kg of body weight. The three remaining groups each received 1% DMSO (negative control), 5 mg/kg chloroquine (positive control), and distilled water (normal control). All doses were administered orally. These mice were treated for 4 days (D1, D2, D3, and D4). From the 5th to the 10th day (D5 to D10), the parasitemia of each mouse was evaluated. Blood smears were prepared from the tail of each mouse, stained with May Grunwald-Giemsa, and observed under a microscope at 100× objective. From the 11th to the 30th day, mice were observed for the average survival rate. Parameters such as weight and temperature were recorded. The parasitemia, as well as the reduction rate, were determined and calculated as follows:

Parasitemia (P):


$$\text{P}=\frac{\text{Number}\;\text{of}\;\text{parasitized}\;\text{reds}\;\text{blood}\;\text{cells}}{\text{Number}\;\text{of}\;\text{total}\;\text{reds}\;\text{blood}\;\text{cells}}\times 100$$

The reduction rate:


$$\text{RR}=\frac{\text{Pa}\text{rasitemia}\;\text{of}\;\text{the}\;\text{negative}\;\text{control}-\text{Parasitemia}\;\text{of}\;\text{the}\;\text{test}\;\text{group}}{\text{Parasitemia}\;\text{of}\;\text{the}\;\text{negative}\;\text{control}}\times 100$$

The average survival rate:


$$\text{ASR}=\frac{\text{Number}\;\text{of}\;\text{remening}\;\text{mice}}{\text{Total}\;\text{number}\;\text{of}\;\text{mice}}\times 100$$

#### 2.4.3 Curative test

**Figure 1:**
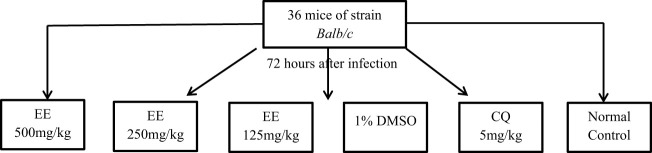
Experimental design.

The curative activity was evaluated by the method of Ryley (Ryley and Peters, 1970) (**Figure 1**). A total of 36 albino mice (male and female) with an average weight of 25 ± 0.5 g were divided into six groups of six mice each. Five groups of these mice were infested intraperitoneally on day (D1) with 1×10^7^ parasitized red blood cells contained in 200 μL. Seventy-two hours after infestation, the experimental animals were treated on D4, D5, D6, and D7 at 24-h intervals for 4 days with the ethanol extract (EE). Groups I, II, and III received the extracts at a dose of 500–250–125 mg/kg. Group IV (Negative Control) received 1% DMSO, Group V (Positive Control) received chloroquine (CQ) at a dose of 5 mg/kg, and Group VI (Neutral Control) received distilled water. On D4, the parasitemia of all experimental groups was determined by May-Grünwald-Giemsa staining.

The average survival rate of the treatment groups was determined arithmetically by calculating the average survival time in days from the day of infestation until the 30th day (D1–D30) (Sidiki et al., 2023). The activity of the extract on parasites was expressed as a function of the reduction in parasitemia of treated mice compared to mice in the control group (Singh et al., 2015). Percentage inhibition and parasitemia were also calculated.

For hematological analyses, three mice per group were anesthetized using cotton soaked in chloroform and sacrificed; the blood obtained was introduced into EDTA tubes for hematological analysis using an automated hematology analyzer (Nihonkohden-celltax). The results were expressed as a mean ± standard deviation.

### 2.5 Quantitative phytochemical screening

The extracts were tested for the presence of flavonoids, alkaloids, triterpenoids, saponins, quinones, phenolic compound, and tannins using standard procedures described by Junaid and Patil (Shaikh and Patil, 2020).

### 2.6 Ethical approval

All authors hereby declare that the “Principles for the Care of Laboratory Animals” (NIH Publication No. 85-23, revised 1985) have been followed, as well as specific national laws, where applicable. All experiments were reviewed and approved by the Department of Animal Biology, Faculty of Science, University of Dschang.

### 2.7 Statistical analysis

The data collected were entered on Microsoft Office Excel version 2010 software. Subsequently, the 50% inhibitory concentrations (IC_50_) were determined using the concentration–response curves obtained by plotting the logarithm of the concentration as a function of the percentage of inhibition using the GraphPad Prism 8 software. The normality test was done using the Shapiro–Wilk test. The Kruskal–Wallis test and one-way ANOVA (analysis of variance) were used to compare the mean developmental inhibition rates of *P. berghei*. The tests were statistically significant at *p* ≤ 0.05.

## 3 Results

### 3.1 *In vitro* antiplasmodial activity

**Table 1** shows the *in vitro* antiplasmodial activity of *E. sigmoidea*. It can be observed that the two extracts—the aqueous and ethanol extracts—inhibited the development of Plasmodium strains (3D7 for the sensitive strain and Dd2 for the resistant strain). For the two strains, the ethanol extract had an IC_50_ of 6.44 ± 0.08 µg/mL in the chloroquine-sensitive strain and an IC_50_ of 7.53 *±* 0.22 µg/mL in the chloroquine-resistant strain. This ethanol extract had a lower IC_50_ than the aqueous extract, which had an IC_50_ of 29.51 ± 3.63 µg/mL in the chloroquine-sensitive strain and an IC_50_ of 35.23 ± 3.17 µg/mL in the chloroquine-resistant strain. The RI was 1.17 and 1.19 for the ethanol and aqueous extracts, respectively. When the RI is less than 1, this means that the drug acts preferentially on the chloroquine-sensitive strain of Plasmodium, whereas when the RI is greater than 1, the drug acts without distinction of Plasmodium strain.

**Table 1:** *In vitro* antiplasmodial activity (IC_50_) of *E. sigmoidea*.

IC50±SD (µg/ml)				
Sample	PfDd2	Pf3D7	RI	Observation
**Aqueous**	35.23 ± 3.17	29.51 ± 3.63	1.19	Active
**Ethanolic**	7.53 ± 0.22	6.44 ± 0.08	1.17	Active
**Artemisinin**	0.043 ± 0.01	0.034 ± 0.005	1.26	NA
**Chloroquine**	0.64 ± 0.08	0.029 ± 0.0004	22.07	NA

NA, Non applicable.

### 3.2 *In vivo* suppressive effect of the ethanol extract on the evolution of parasitemia

**Figure 2:**
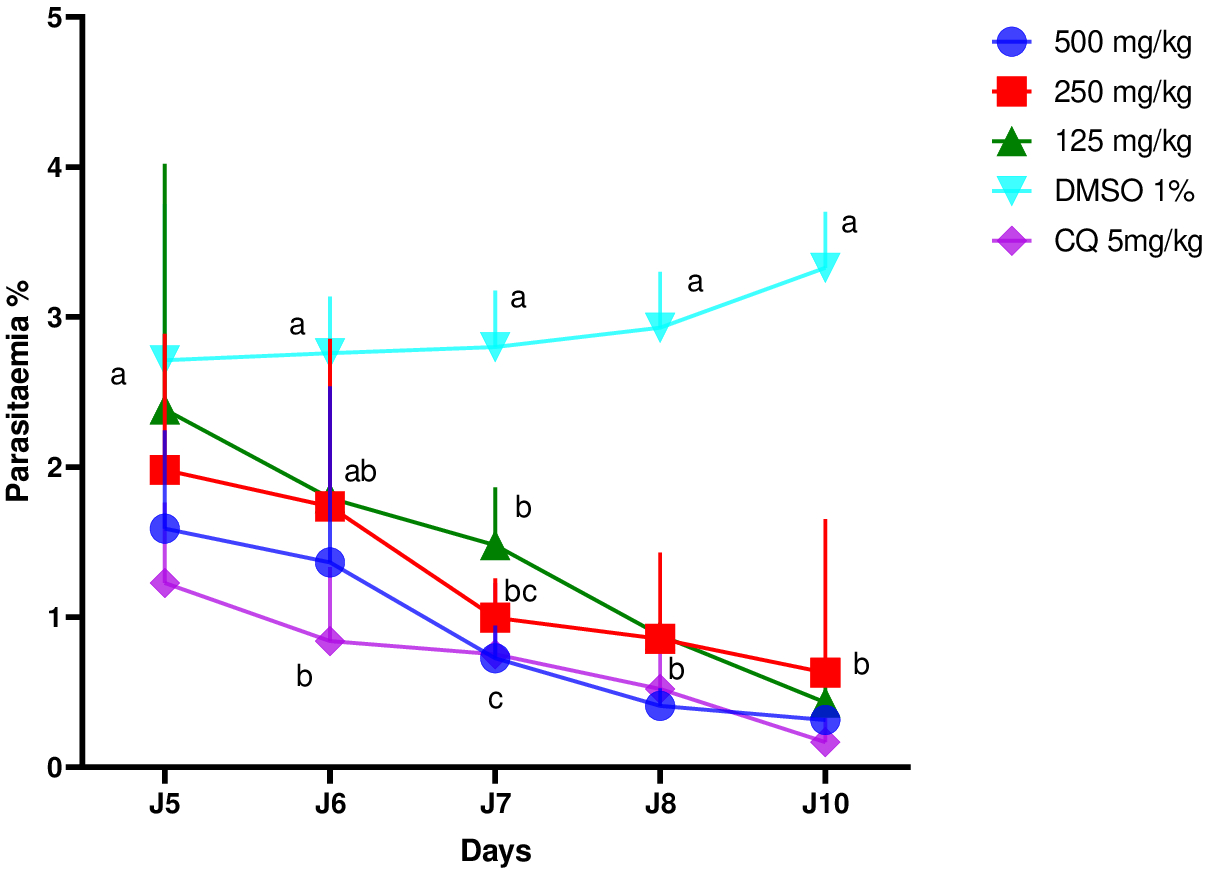
Effects of different doses of the ethanol extract of *Erythrina sigmoidea* on the evolution of parasitaemia in mice with respect to time.

**Figure 2** shows that the ethanol extract in all doses used inhibited parasitemia in a dose-dependent manner. Parasitemia on the 10th day increased significantly in the negative control group (2.8% to 3.5%) while it decreased in the positive control group (1.2% to 0.2%). As for the groups treated with the extract, we observed that their parasitemia decreased at doses of 500 mg/kg (1.6% to 0.5%), 250 mg/kg (2% to 0.8%), and 125 mg/kg (2.4% to 0.6%) of body weight.

#### 3.2.1 Mortality rate of experimental animals after treatments with *E. sigmoidea* of the suppressive test

**Table 2** shows that the groups that received 5 mg/kg of chloroquine and extract of *E. sigmoidea* with doses of 250 mg/kg and 500 mg/kg of body weight had no mortality on the 10th day, whereas the untreated group and the group receiving the extract with a dose of 125 mg/kg had a mortality rate of 16.67%. It is only after the 10th day that we recorded mortality in the groups treated with doses of 250 mg/kg and 500 mg/kg, with mortality rates of 83.33% and 66.67%, respectively, on the 30th day. We recorded mortality of 83.33% in the untreated group, and the group receiving the extract with a dose of 125 mg/kg had a mortality rate of 100% on the 30th day. The survival rate was 100% in the group treated with chloroquine on the 30th day.

**Table 2:** Mortality rate (%) of experimental animals after treatment with different doses of extracts during the suppressive test.

Products	Dosage (mg/kg)	Post treatment period (day)%				
		10	15	20	25	30
1% DMSO		16.67	33.33	50	66.67	83.33
Chloroquine	5	0	0	0	0	0
*Erythrina sigmoidea*	125	16.67	33.33	50	83.33	100
	250	0	16.67	33.33	66.67	83.33
	500	0	16.67	33.33	66.67	66.67

#### 3.2.2 Parasitemia reduction rate during post-treatment period of the suppressive test

**Table 3** shows that the reference drug (chloroquine) used in this study exerts a suppressive activity of 94.89% with a dose of 5 mg/kg of body weight from the 10th day. The extract administered at doses of 500 mg/kg, 250 mg/kg, and 125 mg/kg/day inhibited growth of *P. berghei* by 87.69%, 86.79%, and 81.08%, respectively, in mice on the 10th day.

**Table 3:** Parasitemia reduction rate during post-treatment period of the suppressive test.

Treatment	Reduction rate of parasitaemia (%)				
	D5	D6	D7	D8	D10
500 mg/kg	41.33	50.72	69.64	82.93	87.69
250 mg/kg	26.94	37.32	64.64	69.28	86.79
125 mg/kg	12.18	35.14	46.43	62.46	81.08
1% DMSO	0	0	0	0	0
CQ5 mg/kg	54.98	69.56	79.28	84.64	94.89

D: day.

#### 3.2.3 Effect of *E. sigmoidea* extract on hematological parameters during the suppressive test

**Table 4** presents the effects of ethanol extract on hematological parameters. In the groups treated with plant extracts and chloroquine, parameters such as hemoglobin, red blood cells, and hematocrit increased compared to the group that received DMSO (negative control) and, on the other hand, decreased compared to the neutral control group. The white blood cell value increased while the other measured parameters rather decreased when compared to the normal control. The values of the other parameters of the groups treated with the extract and the chloroquine showed a statistically significant difference (*p* < 0.05) when compared with those of the normal and negative control groups.

**Table 4:** Effect of the extract on the hematological parameters in the suppressive test.

Treatments	RBC	HGB	HCT	MCV	MCH	MCHC	WBC	PLT	LYM	MO	Gra	MPV	RDWCV	RDWSD	PCT	PDW
**500 mg/kg**	8.74 ± 1.57^a^	12.75 ± 1.06^a^	36.7± 4.81	42.15 ± 2.05	14.7 ± 1.41^a^	33.35 ± 0.50	9.15 ± 3.46^a^	323 ± 166.88	44 ± 12.59	9 ± 0.28	45.8 ± 12.87	8.1 ± 1.41	17.25 ± 2.75	29 ± 3.25	0.25 ± 0.08	14.7 ± 1.70
**250 mg/kg**	7.54 ± 1.77^a^	11.6 ± 3.90^a^	32.7 ± 10.89	42 ± 4.38	15.2 ± 1.70^a^	35.4 ± 0.28	33.4 ± 34.51^a^	992 ± 562.86	29.7 ± 0	6.9 ± 0	63.4 ± 0	6.7 ± 0	18.85 ± 0.07	32.25 ± 3.18	0.66 ± 0.37	16.25 ± 1.06
**125 mg/kg**	8.9 ± 1.50^a^	13.7 ± 1.80^a^	40 ± 2.96	44.9 ± 3.46	15.4 ± 2.14^a^	34.3 ± 4.09	8.8 ± 5.40^a^	559 ± 333.17	56.5 ± 6.70	14.6 ± 7.14	28.9 ± 8.31	7.9 ± 6	16 ± 9.27	28.7 ± 9.48	0.44 ± 0.29	13.6 ± 3.11
**1% DMSO**	5.82 ± 1.50^b^	44.55 ± 5.02^b^	15.95 ± 1.77	35.8 ± 0	43.65 ± 0.64^b^	35.8 ± 0	5.45 ± 1.48^b^	661.5± 630.03	58.6 ± 32.31	5.7± 1.34	35.6 ± 30.97	7.05 ± 1.20	17.85 ± 1.76	31.95 ± 6.72	0.43 ± 0.37	15.65 ± 0.59
**CQ 5mg/kg**	9.15 ± 1.34^a^	7.62 ± 0.54^a^	11.85 ± 0.64	33.3± 2.83	15.55 ± 0.21^a^	35.6± 1.13	8.1± 4.95^a^	717± 301.28	25.85 ± 14.07	7.15 ± 2.05	67± 16.12	6.9± 0.28	18.9± 0.56	33± 0.56	0.49 ± 0.18	15.35 ± 0.21
**Normal**	8.40 ± 0.25^a^	13.10 ± 0.70^a^	37.8 ± 1.13	45 ± 0	15.6 ± 0.42^a^	34.65 ± 0.78	7.15 ± 0.78^a^	1,076 ± 190.918	43.6 ± 2.40	17.6 ± 2.97	38.8± 0.56	6.30 ± 0.14	19.25 ± 1.20	34.65 ± 2.19	0.68 ± 0.14	14.8 ± 0.42

RBC, red blood cells; PLT, platelets; MCV, mean corpuscular volume; HBG, hemoglobin; WBC, white blood cells; HCT, hematocrit; MCH, mean corpuscular content in hemoglobin; MCCH, mean corpuscular concentration in hemoglobin; MO, monocytes; Gra, granulocytes; LYM, lymphocytes; MPV, mean platelet volume; PCT, procalcitonin; RDWCV, red cell distribution with coefficient of variation; RDWSD, red cell distribution with standard deviation; PDW, width platelets distribution. For the same column; values carrying the same superscript letter are not significantly different at p ≥ 0.05.

#### 3.2.4 Effect of *E. sigmoidea* ethanol extract on body temperature during the suppressive test

**Table 5** shows the effect of treatment on body temperature. It follows from the analysis of this table that temperature did not really have an impact on the evolution of the suppressive test.

**Table 5:** The effect of treatment on body temperature in the suppressive test.

Treatment	Day 2	Day 3	Day 4	Day 5	Day 6	Day 7	Day 8
500 mg/kg	34.77 ± 1.55^a^	33.55 ± 1.59^b^	34.52 ± 1.20^a^	34.13 ± 1.47^a^	34.33 ± 0.97^a^	34.48 ± 1.22^a^	34.62 ± 1.01^a^
500 mg/kg	35.27 ± 1.15^b^	33.13 ± 0.62^b^	34.15 ± 0.87^a^	34.10 ± 0.93^a^	34.15 ± 1.11^a^	35.12 ± 0.65^b^	35.50 ± 0.40^b^
125 mg/kg	34.15 ± 1.48^a^	33.43 ± 0.92^b^	34.28 ± 0.93^a^	33.74 ± 0.80^b^	33.92 ± 0.89^b^	34.76 ± 1.51^a^	34.87 ± 0.97^a^
1% DMSO	34.23 ± 1.50^a^	34.70 ± 1.11^a^	35.55 ± 0.29^b^	34.27 ± 1.18^a^	35.03 ± 1.07^b^	35.03 ± 1.07^b^	35.52 ± 0.51^b^
CQ 5 mg/kg	34.72 ± 1.15^a^	34.08 ± 1.85^a^	34.40 ± 1.64^a^	34.42 ± 1.25^a^	34.78 ± 1.49^a^	35.65 ± 0.32^b^	34.78 ± 1.27^a^
Normal	34.72 ± 1.15^a^	34.84 ± 1.62^a^	35.22 ± 1.04^b^	33.20 ± 0.43^b^	33.46 ± 1^b^	34.20 ± 1.42^a^	35.20 ± 0.95^b^

The values that carry the same superscript letter are not significantly different at p <0.05.

#### 3.2.5 Effect of ethanol extract of *Erythrina sigmoidea* on body weight during the suppressive test

**Table 6** shows the evolution of weight with respect to time during the suppressive test. It can be observed that on day 8, values of the different masses were slightly modified with a statistically significant difference (*p* < 0.05). However, there is an increase in weight in the positive control group, unlike the other groups.

**Table 6:** Evolution of weight with respect to time during the suppressive test.

Treatment	Day 2	Day 3	Day 4	Day 5	Day 6	Day 7	Day 8
500mg/kg	28.17 ± 2.26^a^	30.50 ± 1.81^a^	29 ± 2.86^a^	27.17 ± 2.90^a^	26.23 ± 3.67^b^	27.60 ± 3.65^b^	28.80 ± 3.11^b^
250mg/kg	31.27 ± 1.50^a^	33 ± 1.60^a^	29.17 ± 2.19^a^	30 ± 2.37^a^	29.18 ± 1.48^c^	30.20 ± 0.84^b^	31.10 ± 0.58^b^
125mg/kg	28.67 ± 3.39^c^	29.67 ± 3.10^c^	28.33 ± 3.35^a^	29.33 ± 3.74^b^	28.62 ± 1.86^c^	29.60 ± 3.13^b^	30.20 ± 4.15^b^
1% DMSO	26.67 ± 1.96^b^	26 ± 1.50^b^	25.33 ± 0.84^b^	24.67 ± 1.79^b^	23.51 ± 1.95^b^	22.75 ± 0.96^b^	20.25 ± 1.71^c^
CQ 5mg/kg	24 ± 1.60^c^	26.5 ± 1.96^c^	25.50 ± 2.06^c^	26.83 ± 1.83^a^	25.46 ± 2.95^c^	27 ± 2.76^c^	28.17 ± 3.54^b^
Normal	22.67 ± 2.14^c^	20.5 ± 2.19^c^	20.17 ± 2.34^c^	20.50 ± 2.73^c^	19.13 ± 2.56^c^	21.7 ± 2.93^c^	24.67 ± 3.01^c^

The values that carry the same superscript letter are not significantly different at p <0.05.

### 3.3 Curative effect of the ethanol extract on the development of *Plasmodium berghei*

**Figure 3:**
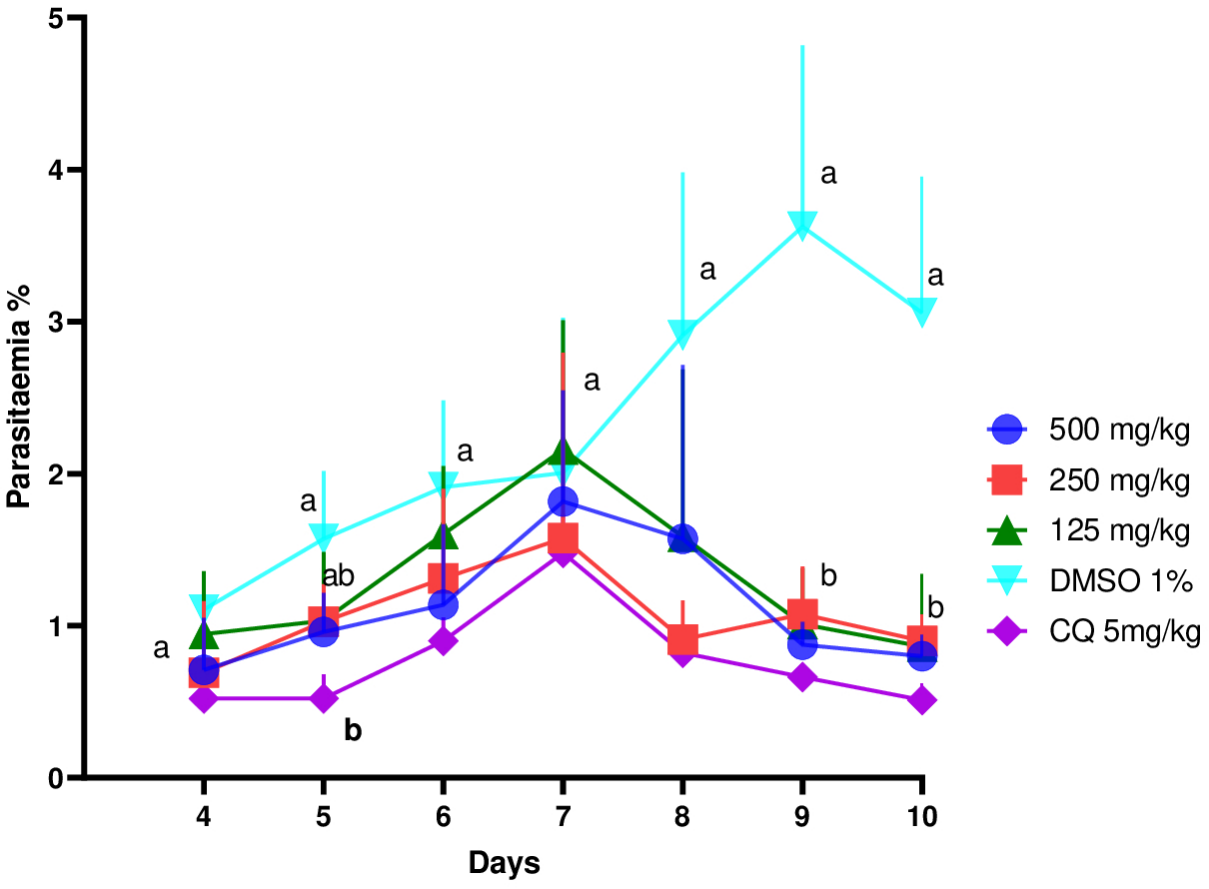
Effect of ethanol extract of *Erythrina sigmoidea* on the evolution of *Plasmodium berghei* parasitaemia in mice with respect to time.

**Figure 3** shows the evolution of parasitemia in mice treated with the ethanol extract of *E. sigmoidea* at doses of 125 mg/kg, 250 mg/kg, and 500 mg/kg. In general, these curves showed a growth phase that corresponded to the multiplication of the Plasmodium after the infection, then an inhibition phase of the development of Plasmodium after the mice had started assimilating the treatments (extracts and chloroquine). On the 10th day, the negative control group had its parasitemia increase (1.2% to 3.8%), while the other groups’ parasitemia dropped considerably.

#### 3.3.1 Mortality rate during post treatment period for the curative test

**Table 7** shows the mortality rate (%) during the post-treatment period of the curative test. It follows from the analysis of this table that the groups receiving chloroquine at 5 mg/kg and the extract at a dose of 500 mg/kg of body weight had no mortality on the 10th day, whereas in the untreated group and the group receiving the extract at doses of 125 mg/kg and 250 mg/kg, the mortality was 83.33%, 50%, and 33.33%, respectively, on day 20. On the 30th day, the mortality rates in the groups treated with chloroquine 5 mg/kg and the extract at 125 mg/kg and 500 mg/kg doses were 33.33%, 100%, and 66.67%, respectively.

**Table 7:** Mortality rate (%) during the post treatment period of the curative test.

Products	Dosage (mg/kg)	Post treatment period (day) %				
		10	15	20	25	30
1% DMSO		33.33	66.67	83.33	100	100
Chloroquine	5	0	0	16.67	33.33	33.33
*Erythrina sigmoidea*	125	16.67	33.33	50	83.33	100
	250	16.67	16.67	33.33	66.67	100
	500	0	16.67	33.33	66.67	66.67

#### 3.3.2 Parasitemia reduction rate during the curative test

**Table 8** shows that the inhibition rate was lower when compared to the inhibition of the suppressive test. The reference drug (chloroquine) used in this study had an inhibition rate of 87.5% from the 10th day. The ethanol extract of *E. sigmoidea* administered at doses of 500 mg/kg, 250 mg/kg, and 125 mg/kg/day inhibited the growth of *P. berghei* at 80%, 78.5%, and 77.5%, respectively, in mice.

**Table 8:** Parasitemia reduction rate during the post-treatment period of the curative test.

Traitement	Reduction rate of parasitaemia (%)				
	D5	D6	D7	D8	D10
500mg/kg	40	40	15,74	46,05	80
250mg/kg	35,6	31,05	26,85	68,73	78,5
125mg/kg	35,6	15,79	7,41	45,01	77,5
1% DMSO	0	0	0	0	0
CQ5mg/kg	62,5	52,63	30,5	72,51	87,5

#### 3.3.3 Effect of *E. sigmoidea* on hematological parameters during the curative test

**Table 9** shows the effect of *E. sigmoidea* on hematological parameters. It appears from this table that hemoglobin, RBCs, and hematocrit levels were higher in the groups treated with plant extracts and chloroquine than in the DMSO-treated (negative control) group, but lower than in the normal control group. When compared to the normal control, the white blood cell value increased while the other evaluated parameters actually declined. When compared to those of the normal and negative control groups, the values of the other parameters in the groups treated with the extract and the chloroquine revealed a statistically significant difference (*p* ≤ 0.05).

**Table 9:** Effect of extract on the hematological parameters in the curative test.

Treatments	HGB	WBC	HCT	MCV	MCH	MCHC	WBC	PLT	LY	MO	GR	MPV	RDWCV	RDWSD	PCT	PDW
**500 mg/kg**	11 ± 0.99^a^	7.97 ± 1.30^a^	34.75 ± 2.47^a^	43.9 ± 4.10^a^	14.05 ± 1.06	31.9 ± 0.56	6.75 ± 3.61^a^	253 ± 39.60	57.7 ± 22.77	14.55 ± 0.21	27.75 ± 22.98	7.5 ± 0.28	17.1 ± 0.85^a^	29.95 ± 1.34^a^	0.19 ± 0.03	16.25 ± 1.06
**250 mg/kg**	10 ± 0.99^a^	7.03 ± 0.34^a^	34.1 ± 3.25^a^	48.45 ± 2.33^a^	14.2 ± 0.71	29.3 ± 0.14	14.85 ± 5.16^a^	586.5 ± 168.9	47.05 ± 45.18	9.05 ± 7.71	43.9 ± 37.47	7 ± 0.14	16.8 ± 0.28^a^	32.6 ± 2.12^a^	0.4 ± 0.11	17.55 ± 0.35
**125 mg/kg**	10.9 ± 5.23^a^	7.46 ± 3.39^a^	32.95 ± 13.08^a^	44.75 ± 2.76^a^	14.5 ± 0.42	32.5 ± 2.97	9.9 ± 1.41^a^	545.5 ± 267.9	67.8 ± 1.55	5.65 ± 0.92	26.55 ± 0.64	6.85 ± 0.64	16.9 ± 0.99^a^	30.3 ± 3.68^a^	0.3 ± 0.15	16.5 ± 1.41
**1% DMSO**	8.05 ± 2.76^b^	5.26 ± 2.67^b^	27.7 ± 7.64^b^	56.25 ± 14.07	16.05 ± 2.90	28.8 ± 1.98	55 ± 18.53^b^	421 ± 118.8	73.65 ± 7	9.55 ± 9.40	16.8 ± 2.40	7.6 ± 0.99	21 ± 5.37^b^	48.8 ± 23.90^b^	0.3 ± 0.05	16.1 ± 0.28
**CQ 5mg/kg**	10.8 ± 1.83^a^	7 ± 0.28^a^	32.55 ± 1.91^a^	46.45 ± 0.92^a^	15.4 ± 1.98	33.05 ± 3.75	25.15 ± 25.81^a^	1047 ± 175.36	70.15 ± 0.92	12.5 ± 5.51	17.35 ± 6.43	7 ± 0.14	17.45 ± 1.63^a^	32.45 ± 3.60^a^	0.73 ± 0.11	16.15 ± 0.35
**Normal**	11.1 ± 1.13^a^	7.62 ± 1.29^a^	32.65 ± 4.45^a^	43 ± 1.41^a^	14.7 ± 0.99	34.05 ± 1.20	17.05 ± 14.35^a^	763 ± 1.40	61.65 ± 15.91	18.7 ± 16.97	19.65 ± 1.06	6.3 ± 0.14	16.9 ± 1.27^a^	29.1 ± 3.11^a^	0.47 ± 0.08	17.85 ± 0.50

RBC, red blood cells; PLT, platelets; MCV, mean corpuscular volume; HBG, hemoglobin; WBC, white blood cells; HCT, hematocrit; MCH, mean corpuscular content in hemoglobin; MCCH, mean corpuscular concentration in hemoglobin; MO, monocytes; GR, granulocytes; LY, lymphocytes; MPV, mean platelet volume; PCT, procalcitonin; RDWCV, red cell distribution with coefficient of variation; RDWSD, red cell distribution with standard deviation; PDW **=** width platelets distribution. For the same column; values carrying the same superscript letter are not significantly different at p ≥ 0.05.

#### 3.3.4 Effect of *E. sigmoidea* ethanol extract on body temperature during the curative test

**Table 10** shows that the values for temperature were slightly modified with a statistically significant difference (*p* < 0.05). However, animals with the highest temperatures were those of the normal control groups and those of the doses of 250 and 500 mg/kg of body weight on day 8.

**Table 10:** Effect of *E. sigmoidea* ethanol extract on temperature during the curative test.

Treatment	Day 2	Day 3	Day 4	Day 5	Day 6	Day 7	Day 8
500mg/kg	34.55 ± 1.46^a^	35.58 ± 0.43^b^	35.12 ± 1.77^b^	34.55 ± 1.25^a^	34.63 ± 1.92^a^	34.56 ± 2.01^a^	35.16 ± 1.67^b^
250mg/kg	34.60 ± 1.38^a^	35.58 ± 0.42^b^	35.57 ± 1.17^b^	34.42 ± 1.54^a^	32.90 ± 0.48^c^	36.22 ± 0.26^c^	35.20 ± 1.87^b^
125mg/kg	34.15 ± 1.48^a^	35.88 ± 0.36^b^	34 ± 1.83^a^	35.08 ± 0.78^b^	33.30 ± 1.48^c^	35.02 ± 1.47^b^	34.96 ± 1.49^a^
1% DMSO	34.23 ± 1.50^a^	35.82 ± 0.27^b^	35.54 ± 0.82^b^	35.08 ± 1.47^b^	35.90 ± 1.08^b^	35.22 ± 1.44^b^	34.65 ± 1.68^a^
CQ 5mg/kg	34.72 ± 1.15^a^	35.36 ± 0.88^b^	35.12 ± 1.14^b^	35.23 ± 1.25^b^	33.65 ± 1.84^c^	35.40 ± 1.57^b^	34.58 ± 1.81^a^
Normal	34 ± 1.84^a^	35.76 ± 0.37^b^	36.15 ± 0.43^c^	34.37 ± 1.57^a^	35.32 ± 1.51^b^	35.90 ± 0.30^b^	36.03 ± 0.15^c^

The values that carry the same superscript letter are not significantly different at p <0.05.

#### 3.3.5 Effect of ethanol extract of *Erythrina sigmoidea* on body weight during the curative test

**Table 11** shows that on day 8, the values of the different weights were slightly modified with a statistically significant difference (*p* < 0.05). However, there was an increase in weight in the positive control group, unlike the other groups.

**Table 11:** Effect of the curative test on weight.

Treatment	Day 2	Day 3	Day 4	Day 5	Day 6	Day 7	Day 8
500mg/kg	24.50 ± 2.26^a^	24.50 ± 2.81^a^	24.17 ± 2.86^a^	24 ± 2.90^a^	23.67 ± 3.67^b^	23.60 ± 3.65^b^	21.80 ± 3.11^b^
250mg/kg	24.67 ± 1.50^a^	24.17 ± 1.60^a^	24 ± 2.19^a^	23 ± 2.37^a^	25.20 ± 1.48^c^	23.20 ± 0.84^b^	22.33 ± 0.58^b^
125mg/kg	25.33 ± 3.39^c^	25 ± 3.10^c^	24 ± 3.35^a^	24 ± 3.74^b^	25.33 ± 1.86^c^	23.60 ± 3.13^b^	23.20 ± 4.15^b^
1% DMSO	23.67 ± 1.96^b^	23.33 ± 1.50^b^	23.20 ± 0.84^b^	21.80 ± 1.79^b^	23.40 ± 1.95^b^	21.75 ± 0.96^b^	22.25 ± 1.71^b^
CQ 5mg/kg	26.17 ± 1.60^c^	25.67 ± 1.96^c^	25.67 ± 2.06^c^	24.17 ± 1.83^a^	25.50 ± 2.95^c^	27 ± 2.76^c^	27.17 ± 3.54^c^
Normal	25.83 ± 2.14^c^	26 ± 2.19^c^	25.67 ± 2.34^c^	26.33 ± 2.73^c^	26.83 ± 2.56^c^	25.17 ± 2.93^c^	24.67 ± 3.01^a^

The values that carry the same superscript letter are not significantly different at p <0.05.

### 3.4 Qualitative phytochemical screening

**Table 12** shows the phytochemical screening of the ethanol extract of *E. sigmoidea* stem bark. It follows from the analysis of this table that this extract contains flavonoid, phenolic compound, tannin, and saponins.

**Table 12:** Phytochemical screening of *Erythrina sigmoidea* ethanol extract.

Extract	Alkaloids	Flavonoids	Phenolic	Tannin	Triterpenoids	Quinones	Saponines
Ethanol	–	+	+	+	−	–	+

+: present and −: absent.

## 4 Discussion

The IC_50_ values obtained with extracts of *E. sigmoidea* against *P. falciparum* strain using the Trager and Jensen (1997) method show that the extracts have promising activity with IC_50_ 6.44 ± 0.08 µg/mL on the sensitive strain and 7.53 ± 0.22 µg/mL on the resistant strain of the ethanol extract and moderate activity with IC_50_ 29.51 ± 3.63 µg/mL on the sensitive strain and 35.23 ± 3.17 µg/mL on the resistant strain for the aqueous extract. According to WHO guidelines, antiplasmodial activity is classified as follows: highly active for IC_50_ values of 5 g/mL; promising for values of 5–15 g/mL; moderate for values of 15–50 g/mL; and inactive for values greater than 50 g/mL (Philippe et al., 2005). The highest activity was observed with the chloroquine-sensitive strain with an IC_50_ of 0.029 ± 0.0004 µg/mL and 0.64 ± 0.08 µg/mL on the resistant strain. These findings are consistent with those of Nadia et al. (2020), who tested *Bidens pilosa* and found it to be highly active against the chloroquine resistant strain. This difference observed between the antiplasmodial activity of the two extracts could be linked to the difference in the Plasmodium strains on which the extracts were evaluated and also to the chemical composition of the plant, which may vary according to certain characteristics such as climatic and edaphic factors (Wabo et al., 2011). This could also be explained by the nature of the solvent used for the extraction. According to Koagne et al. (Houari, 2006), flavonoids could lead to more useful derivatives for the development of an antiplasmodial agent and have antiplasmodial properties.

The level of parasitemia was reduced in a dose-dependent manner, which is an indicator of the antiplasmodial activity of *E. sigmoidea*. These percentages of inhibition were considered very active according to the classification of Munoz et al. (2000), which stipulates that an extract is considered active if its inhibition is ≥30%. This observation was also reported by Zaruwa et al. (2018) when evaluating the suppressive activity of the ethanol extract of *Achyranthes aspera* and *Ficus thoningii* on the development of *P. berghei* on “*Swiss*” mice. The reduction of parasitemia was also reported by Kaur et al. (2021) on the effect of the hydroethanolic extract of *B. variegata* on *Leishmania donovani.* Similarly, Olasehinde et al. (2016) in Nigeria evaluated the suppressive activity of the ethanol extract of *Moringa oleifera* on the model of mouse *M. musculus*. On the other hand, Camara (2020) presented a weak inhibition of development of *P. berghei* with the ethanol extracts of *Terminalia albida* from Kankan and *Rourea minor*. Inhibition was 13% for *R. minor* at 65 mg/kg and 14% for *T. albida* Kankan at 100 mg/kg. This low inhibition could be due to the diversity of molecules present in plants. The phytochemical constituents might have acted by destroying the parasite cytosol, membrane, mitochondria, and digestive vacuole of the parasites.

The negative control recorded the highest mortality rate (83.33%). Our results are in line with those obtained by Camara (2020) when evaluating the suppressive activity of *D. velutinum* extracts and *R. minor* on *P. berghei* with greater mortality in the negative control with 100% death on the 8th day. Similarly, Adebajo et al. (2014) in the suppressive test also recorded greater mortality in the untreated group. This could be justified by the fact that the negative control group is the infected and untreated group.

A significant difference (*p* < 0.05) was seen in weight between the treatment groups and the control group. When compared to the untreated group, the treated groups lost less weight. These findings support those made by Sulaiman et al. (Rukayyah et al., 2015). A little weight gain was observed in the groups that got the extract at various doses during the post-treatment period. This shows that this plant might have compounds that are useful for treating malnutrition in children and preventing vitamin deficits. Our results are similar to those of Camara (2020) when evaluating the weight of animals after treatment with the *Desmodium velutinum* extract and *R. minor* during the suppressive test as well as the work of Ma et al. (2011) who also presented *D. velutinum* (Fabaceae) as a plant fighting against nutritional deficiencies.

Hematological parameters are reliable parameters for assessing the state of health. White blood cells play a role in immune defense against foreign bodies typically through leukocytosis and antibody production in infected and untreated animals. This observation was made by Noumedem et al. (Nadia et al., 2020) with *B. pilosa* where the average white blood cell count was higher in the untreated group compared to the other treated groups. The anemia observed in infected and untreated animals could be due to the destruction of red blood cells, itself caused by the multiplication of the parasite. The rate of hematocrit is directly related to anemia. This result corroborates with those of Okombé (Okombe et al., 2011) obtained with the powder of *Vitex thomasii* and indicates that the products help animals withstand the effects of parasitism.

During the curative test, the inhibitions were lower compared to the inhibitions obtained in the suppressive test. During the 3 days without treatment, the plasmodia may have developed and adapted better to their environment. No inhibition was obtained in the untreated group. Also, Omagha et al. (2021) also demonstrated inhibition of the parasitemia with a mixture of two cocktails of plants commonly used in southwestern Nigeria. This justifies their use in the treatment of malaria in Western Cameroon. This inhibition may be due to the diversity of molecules present in plants.

The mortalities recorded in the groups receiving the treatments are not totally linked to the hyperparasitemia but to an immunopathology that promotes the accumulation of immune cells in the brain and the dysfunction of the organ and leads to symptoms such as paralysis and head deviation; this is in line with the results obtained by Musila et al. (2013), who recorded 100% death in the untreated group on the 10th day. On the 25th day, we recorded 100% mortality in the untreated group and the group that received the extract at a dose of 125 mg/kg. This is due to the resurgence of the parasite due to a low dose of the extract administered. This result corroborates those found by Haidara et al. (2016). This would be justified by the fact that the negative control group is the infected and untreated group.

## 5 Conclusion

The present study substantiates local claims about the efficacy of *E. sigmoidea* in the treatment of malarial infection. The ethanol extract of *E. sigmoidea* has showed *in vitro* and *in vivo* antimalarial properties and have a higher reduction rate of parasitemia on the inhibition of the development of *P. berghei* NK65 strain, which justifies its use in traditional medicine for the treatment of malaria. However, it will be necessary to evaluate the safety of *E. sigmoidea* in order to assess its toxicity.

## Data availability statement

The raw data supporting the conclusions of this article will be made available by the authors, without undue reservation.

## Ethics statement

All authors hereby declare that the "Principles for the Care of Laboratory Animals" (NIH Publication No. 85-23, revised 1985) have been followed, as well as specific national laws, where applicable. All experiments were reviewed and approved by the Department of Animal Biology, Faculty of Science, University of Dschang.

## Author contributions

TS: Conceptualization, Investigation, Writing – original draft. NN: Conceptualization, Supervision, Writing – review & editing. YC: Conceptualization, Supervision, Writing – review & editing. GG-A: Data curation, Investigation, Writing – original draft. MA: Conceptualization, Investigation, Writing – original draft. NS: Data curation, Investigation, Methodology, Writing – original draft, Writing – review & editing. TK: Conceptualization, Data curation, Investigation, Writing – original draft. VP: Methodology, Supervision, Writing – review & editing. HH: Data curation, Methodology, Supervision, Conceptualization, Writing – original draft, Writing – review & editing.
